# Recent Progress in Optically-Active Phthalocyanines and Their Related Azamacrocycles

**DOI:** 10.3389/fchem.2020.595998

**Published:** 2020-10-19

**Authors:** Yusuke Okada, Tomonori Hoshi, Nagao Kobayashi

**Affiliations:** ^1^Faculty of Textile Science and Technology, Shinshu University, Ueda, Japan; ^2^Clinical Research, Innovation and Education Center, Tohoku University Hospital, Sendai, Japan

**Keywords:** chiral, optically active, phthalocyanine, binaphthyl, circular dichroism, porphyrin

## Abstract

Optically-active phthalocyanines (Pcs) and related macrocycles reported in the 2010–2020 period are introduced in this review. They are grouped into several categories: (1) chiral binaphthyl-containing Pcs, (2) optically active alkyl chain-containing Pcs, (3) chiral axial ligand- coordinated or -linked Pcs, (4) chiral subphthalocyanines (SubPcs), and (5) related azamacrocycles. For each compound, the structure and important characteristics are summarized.

## Introduction

Optically-active or chiral macrocycles of azaporphyrinoids have attracted the attention of synthetic and theoretical chemists, as well as researchers in the fields of applied chemistry and physics, and natural science (Kadish et al., 2003a). Although some small chiral molecules including porphyrins have been studied due to their relatively easy synthesis and specific optical, medicinal, and catalytic properties (Kadish et al., 2000, 2003b), optically active azaporphyrins including phthalocyanines (Pcs) have mainly been introduced by researchers who want to have intense CD signal in the visible region, since azaporphyrins have theoretically more intense absorption band than normal porphyrins in the visible region (the so-called Q band). In the twenty-first century, several review articles have already been published (Kobayashi, 2001, 2010, 2012; Lu and Kobayashi, 2016). However, in this review, we summarize the reports on chiral Pcs and their related macrocycles published in the last 10 years, between 2010 and 2020. After collecting papers on chiral, i.e., optically-active Pcs and related macrocycles, we observed that they are roughly grouped into five categories: (1) chiral binaphthyl (Binap) and related aromatic molecule-containing Pcs, (2) optically-active alkyl chain-containing Pcs, (3) chiral axial ligand-coordinated or -linked Pcs, (4) chiral subphthalocyanines (SubPcs), and (5) related azamacrocycles. Papers containing chiral binaphthyls number more than half of these publications, and this may be due to several factors: easy preparation of binaphthyl-linked precursors, very little possibility of racemization under the experimental conditions, and an expected large circular dichroism (CD) intensity. Reports on optically-active alkyl chain-containing Pcs are also frequently seen, and these are generally used to examine liquid crystalline properties, since long alkyl chain (generally more than 10 carbon)-containing Pcs are known to form liquid crystals (= mesophase) by varying the temperature. Other interesting azamacrocycles which can be congeners of or related to Pcs have also appeared from several groups across the world. We introduce these in order in this review.

## Binap and Binap-Related Substituent-Linked Systems

### Synthesis

**Figure 1:**
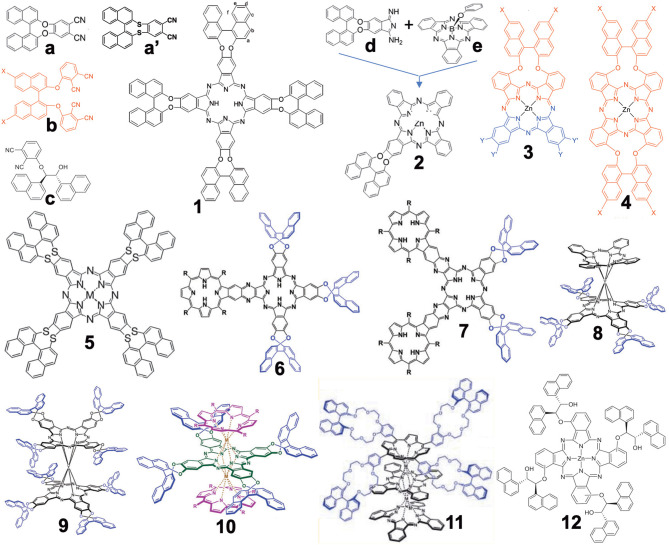
Structures of precursors (**a–e**) and Binap-containing Pcs (**1–12**). In the case of 3D structures (**6–11**), only *R*-enantiomer structures are shown.

Figure 1 summarizes the structures grouped into this category (in the case of 3D-structures, **6–11**, only *R*-enantiomers which have an anti-clockwise binaphthyl structure are shown). In 2012, the synthesis of compound **1** from precursor **a** was reported (Wang et al., 2012). Previously, similar compounds **4** had been reported from a precursor containing sulfur instead of oxygen in **a** (i.e., **a′**) (Kobayashi et al., 1999a) due to the then-known low racemization probability of **a'** under the experimental conditions. However, it was possible to prepare **a** from commercially available 2,2′-dihydroxybinaphthyl and 4,5-dichlorophthalonitrile in one step without racemization, while the cyclic tetramerization step leading to **1** was also free from racemization. Accordingly, both **a** and **1** were later used in chiral Pc chemistry. For example, the zinc complex of **1** could form a cofacial dimer by stepwise coordination of two quinuclidine molecules (Giménez-Agulló et al., 2016), which was confirmed by the change of absorption spectra and ^1^H-NMR signal. In addition, **1** was used to construct chiral bis(phthalocyaninato)yttrium double-decker complexes **8** and **9** (Zhou et al., 2014), as well as a porphyrin-Pc heteroleptic rare-earth triple decker complex **10** (Wang et al., 2014). These were synthesized by metal-free **1** and Y(acac)_3_·*n*H_2_O or **1** and metal-free porphyrin or Pc in the presence of Y(acac)_3_·*n*H_2_O or Dy(acac)_3_·*n*H_2_O, followed by purification using column chromatography.

Compound **2** was obtained by ring-expansion reaction of a SubPc with isoindole derivative **d** which was obtained by bubbling ammonia gas to dinitrile **a** (Zhao et al., 2015). The ring-expansion using SubPc and isoindoline derivatives *per se* was introduced in 1990 as a means to obtain mono-substituted ABBB-type Pcs (Kobayashi et al., 1990, 1999b). Since then, more than 300 papers have appeared on the use of this method (Claessens et al., 2002, 2014; Kobayashi, 2002). Compounds **3** and **4** containing long alkyl chains were prepared by Torres' group in order to study their aggregation (Revuelta-Maza et al., 2019). The precursor **b** was reported as early as 1998 as a stable phthalonitrile which does not racemize under normal Pc formation conditions (Kobayashi, 1998), so that the reaction of **b** and long-alkyl chain-containing phthalonitrile afforded Pc **3** preferentially, and reactions of only **b** produced **4** in relatively high yield.

Compounds **6** and **7** contain porphyrin units. In order to synthesize these compounds, mixed condensation between **a** and pyrazinonitrile fused to a trimethylphenylporphyrin was performed in the presence of lithium in refluxing pentanol, and **6** and **7** were separated after purification using column chromatography in ca. 6–10% yield (Zhang et al., 2017). Triple-decker compound **11** containing both porphyrin and two Pc units were prepared in ca. 40% yield by reacting the corresponding metal-free crowned binaphthyl-linked porphyrin and substituent-free H_2_Pc in the presence of a rare-earth metal, Mt(acac)_3_·*n*H_2_O (Mt = Eu, Y), in refluxing trichlorobenzene (Lu et al., 2011). Although the reaction temperature was 218°, no racemization of the binaphthyl unit occurred.

Compound **12** was synthesized from precursor **c** which was prepared from 3-nitrophthalonitrile and *(1R,2R)*-1,2-di(naphthalen-1-yl) ethane-1,2-diol in the presence of Zn(OAc)_2_·*n*H_2_O and a catalytic amount of DBU (1,8-dia-zabicyclo[5.4.0]undec-7-ene) by a Turkish group (Gok et al., 2018).

### Spectroscopic Properties

**Figure 2:**
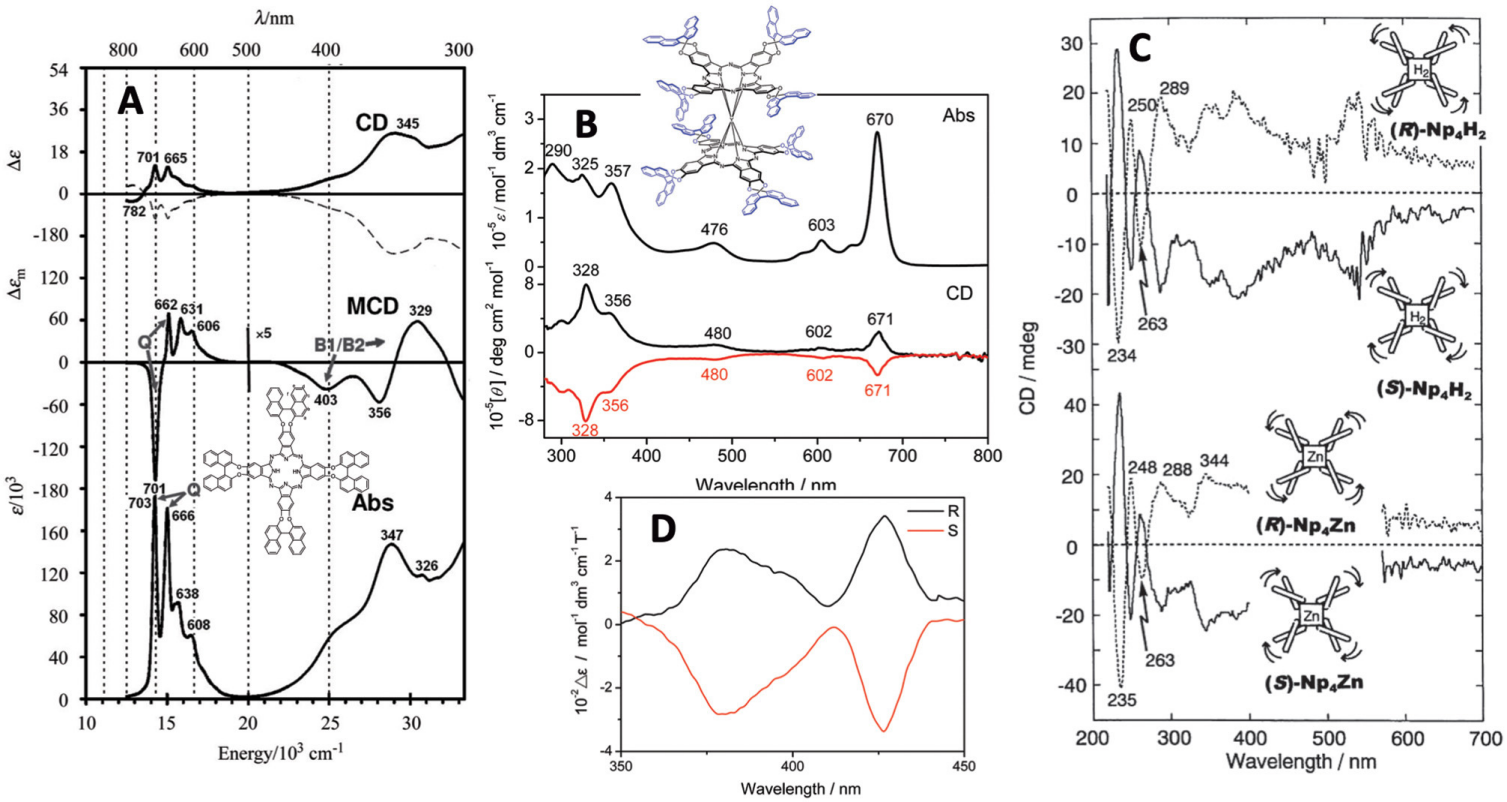
**(A)** Absorption, MCD and CD spectra of **1** [solid and broken lines are for *(R)-* and *(S)-*enantiomer, respectively], **(B)** Absorption and CD spectra of **9** [black and red lines are for *(R)-* and *(S)-*enantiomer, respectively], **(C)** FDCD (ca. 220–270 nm) and FDICD spectra (ca. 270–700 nm) for metal-free (top) and zinc (bottom) derivatives of **5**, and **(D)** magnet-chiral dichroism spectra of **10**. Adapted from Kobayashi (2012) and Wang et al. (2012, 2014).

It is important to show at least the electronic absorption, circular dichroism (CD), and magnetic circular dichroism (MCD) spectra of representative compounds. Figure 2 shows some spectra of compounds **1**, **5**, **9**, and **10**. The absorption and MCD spectra of **1** are typical of those of metal-free Pcs, in that it shows four Q-band peaks and Faraday B-term MCD (Fukuda and Kobayashi, 2010). *(R)*- and *(S)*-enantiomers show mainly positive and negative CD, respectively, similarly to **5** (Kobayashi et al., 1999a). A small negative CD trough observed at 782 nm for the *(R)*-enantiomer suggests that **1** is not completely planar. **9** is a cofacial rare-earth double decker. The interpretation of the spectra of these types of double-decker Pcs and porphyrins was already reported in 2005 (Muranaka et al., 2005). Similarly to **1**, *(R)*- and *(S)*-enantiomers show positive and negative CD, respectively, associated with each absorption peak. Although the apparent absorption coefficient of the Q band is larger than that in the Soret region, inversely, the Soret band CD intensity is much larger than the Q band CD intensity, which is due to the energy difference in the binaphthyl absorption (ca. 220–250 nm) and the Soret (ca. 330–350 nm) or Q band (ca. 670 nm). Here, since the induced CD intensity is proportional to 1/($\nu_{\text{N}}^{2}$_−_ $\nu_{\text{Pc}}^{2}$), where ν_N_ and ν_Pc_ are the frequencies of the absorption of naphthalene and Pc, respectively, the Soret band CD becomes stronger than the Q band CD (Kobayashi et al., 2012).

The curves in Figure 2C are both the fluorescence-detected CD (FDCD) (ca. 200–270 nm) and FD “*induced”* CD (FDICD) spectra (ca. 270–700 nm) observed for the metal-free and zinc complexes of **5**, of which the latter FDICD data are the only published data for any compound (Kobayashi, 2012). The FDICD signal was positive for *(R)*-binaphthayl linked species (left-handed conformer), while negative for *(S)-*binaphthayl linked Pcs, suggesting that the (*S*) species (right-handed conformer) absorbs right-circularly polarized light more than the left, and *vice versa* for the (*R*) species (left-handed conformer).

The spectra in Figure 2D are the rare magnet-chiral dichroism spectra of **10** in the 360–450 nm region where strong ICD and MCD signs were detected. The value of Δε (= 10^2^ mol^−1^dm^3^cm^−1^T^−1^), was comparable to those reported previously in porphyrinoids (Kitagawa et al., 2011, 2012).

Comparing the absorption spectra of **6** and **7**, the Q band of **6** splits, while that of **7** does not, as proven by group theory (Kobayashi and Konami, 1996). In these cases, the absorptions are roughly expressed as a summation of the spectra of porphyrin and Pc, but the Q band region is mainly the contribution from the Pc moiety. Viewing from the Pc structure, **6** is mono-substituted type Pc, while **7** is adjacent-type di-substituted Pc, so that Q the band of Pc **6** splits. In both cases, the *(R)-* and *(S)-*enantiomer showed mainly positive and negative CD, respectively. The Q-band MCD of **7** is dispersion type pseudo Faraday A term, since the splitting of the Q_x_ and Q_y_ bands is small. The CD spectra of **11** are noisy, due to the long distance of the perturber (binaphthyl units) from the macrocyclic chromophore. Faraday A-term MCD curves were observed corresponding to each absorption peak, since the excited state of this type of porphyrinoid is orbitally doubly degenerate (Muranaka et al., 2005). Compound **12** was prepared only from 3-((1′*R*,2′*R*)-2-hydroxy-1′,2′-dinaphtylethoxy)phthalonitrile **c**, and no Pc from the corresponding (1′*S*,2′*S*)-enantiomer was reported. Its absorption spectrum is normal, similar to that of regular Pcs, and weak negative CD envelopes were detected associated with the Q and Soret bands.

## Optically-Active Alkyl Chain-Containing Phthalocyanines

### Synthesis

**Figure 3:**
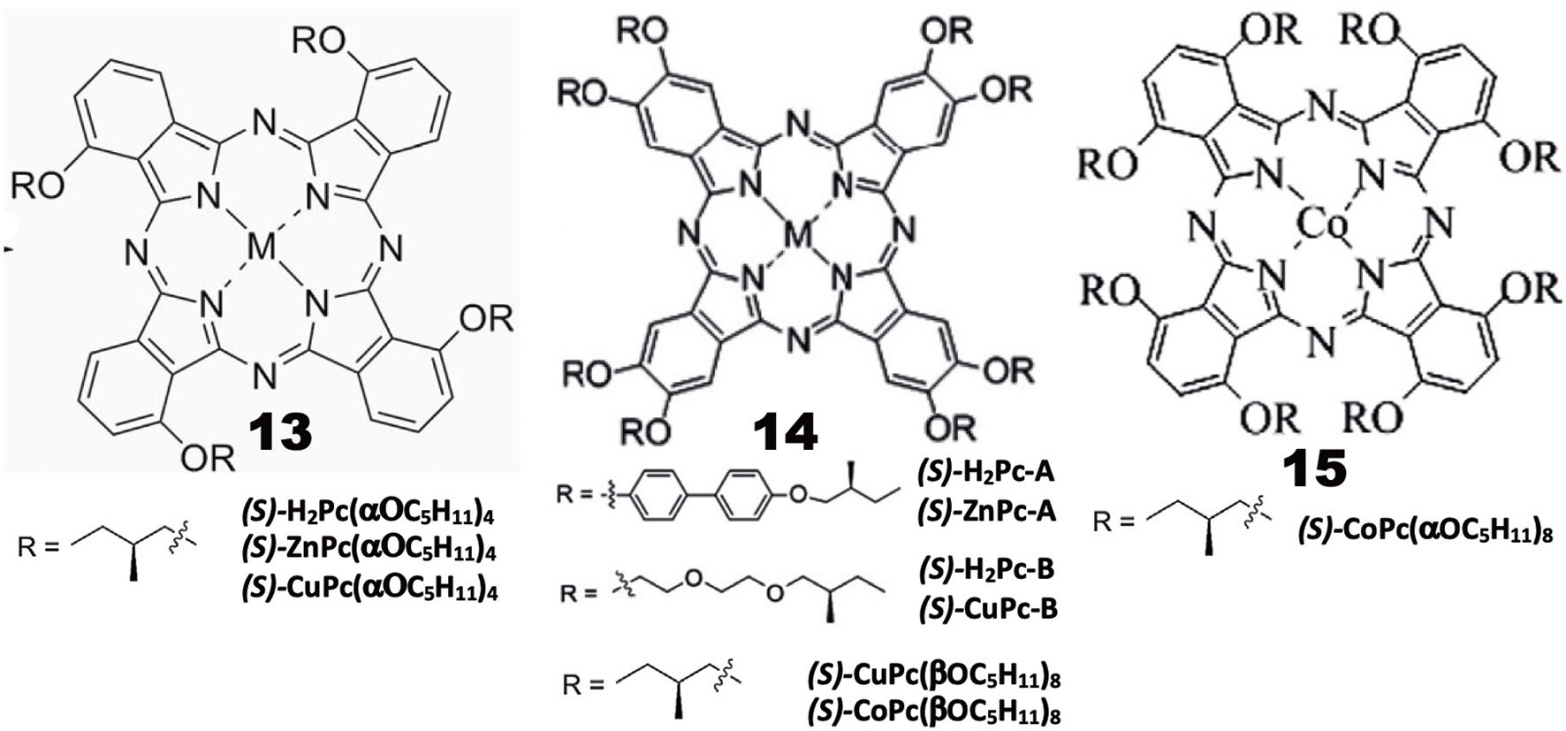
Structures of optically-active alkyl chain-containing phthalocyanines.

Chiral alkyl chain-containing Pcs have often been prepared in order to examine their liquid crystalline properties (Ohta et al., 2011; Basova et al., 2016). The structures of Pc compounds categorized in this group are shown in Figure 3, which are all reported from Zhang's group. As seen in the figure, all substituents contain chiral (*S*)-2-methylbutoxy moieties next to the Pc skeleton or at the outer end of substituent groups. The reaction of 3-nitorophthalonitrile with (*S*)-2-methylbutanol in the presence of K_2_CO_3_ produced 3-((*S*)-2-methylbutoxy)phthalonitrile, whose cyclic tetramerization performed using lithium or in the presence of Zn(OAc)_2_·2H_2_O or Cu(OAc)_2_·*n*H_2_O and DBU produced **13(***(S)*-**H**_**2**_**Pc**-, **13(ZnPc**-, or **13(CuPc(α-OC**_**5**_**H**_**11**_**)**_**4**_**)**, respectively (Tian et al., 2013a).

A similar reaction between *(S)*-4-hydroxy-4′-(2-methylbutoxy)biphenyl and 4,5-dichloro-1,2-dicyanobenzene in the presence of K_2_CO_3_ in DMF produced 1,2-dicyano-4,5-bis[*(S)*-4′-(2-methylbutyoxy)biphenyl]benzene, which was then tetramerized similarly, to yield octa-substituted H_2_- and ZnPcs at β-positions, i.e., **14**(*(S)-***H**_**2**_**Pc-A)** and **14**(*(S)*-**ZnPc-A)** (Zhang et al., 2013). **14**(*(S)-***H**_**2**_**Pc-B)** and **14**(*(S)*-**CuPc-B)** were similarly prepared using a phthalonitrile having a long alkyl chain at the 4,5-positions, which was synthesized in four steps starting from catechol (Tian et al., 2013b). **14**(*(S)***-Cu-** and -**CoPc(βOC**_**5**_**H**_**11**_**)**_**8**_**)** were synthesized using 4,5-di-((*S*)-2-methylbutoxy)phthalonitrile that was prepared by reacting (*S*)-2-methylbutanol with 4,5-dichlorophthalonitrile. **15(CoPc(αOC**_**5**_**H**_**11**_**)**_**8**_**)** was prepared using 3,6-di-((*S*)-2-methylbutoxy)phthalonitrile which was prepared by reaction between (*S*)-2-methylbutanol and 3,6-dihydroxyphthalonitrile (Lin et al., 2015).

### Properties

Induced CD spectra due to optically-active carbon is generally very weak (Kobayashi et al., 2012), and is not well-reproduced by theoretical calculations. Indeed, although most of the Pc compounds in Figure 3 showed weak, noisy CD signals in the Q-band region, their sign is different from compound to compound, irrespective of containing only (*S*)-2-methylbutoxy moieties, i.e., **13(***(S)*-**MtPc(αOC**_**5**_**H**_**11**_**)**_**4**_**)** (Mt = H_2_, Zn, Cu), **14(***(S)***-CuPc(αOC**_**5**_**H**_**11**_**)**_**8**_**)**, **14(***(S)*-**H**_**2**_**Pc-A)**, **14(***(S)-***ZnPc-A)**, **14(***(S)****-*Cu-** and **14(***(S)*-**CoPc(βOC**_**5**_**H**_**11**_**)**_**8**_**)**, exhibited a positive sign, and the intensity of **15(***(S)*-**CoPc(αOC**_**5**_**H**_**11**_**)**_**8**_**)** is almost zero, while both **14(***(S)***-H**_**2**_**-** and **14(***(S)***-ZnPc-B)** showed a negative sign in dilute solutions where aggregation can be neglected. The absorption and CD spectra of aggregates became broader and the CD intensity is stronger than in solution. However, no interpretation is given. It is a pity that the authors did not try similar experiments using compounds containing (*R*)-2-methylbutoxy groups. It is interesting to examine whether the CD of (*R*)-enantiomers always produce signals having a mirror image relationship to those of (*S*)-enantiomers, particularly since Pc compounds containing only (*S*)-2-methylbutoxy groups show both positive and negative CD signs depending on the system.

Investigation of the self-assembled **13(**(*S*)-**H**_**2**_**Pc-** and **13(**(*S*)-**ZnPc(α-OC**_**5**_**H**_**11**_**)**_**4**_**)** by IR and X-ray photoelectron spectroscopies (XPS), and by transmission electronic- (TEM) and scanning electronic (SEM) microscopies, revealed that the metal-free **13(**(*S*)-**H**_**2**_**Pc(α-OC**_**5**_**H**_**11**_**)**_**4**_**)** gets together to form a nanocube, while its zinc derivative, **13(**(*S*)-**ZnPc(α-OC**_**5**_**H**_**11**_**)**_**4**_, gets together to produce assembly of helical nanobelts, which implies that the morphology and handedness of the self-assembled nano-structures are affected by metal coordination (Tian et al., 2013a). Similarly, with respect to **14(**(S)-**H**_**2**_**Pc-A)** and **14(**(S)-**ZnPc-A)**, it was found that the molecular packing in the nano-structures is influenced by the metal-coordination bond, resulting in nano-structured assembly with different morphologies, ranging from nanosheet for **14(**(*S*)**-H**_**2**_**Pc-A)** to helical nanofibers for **14(**(*S*)-**ZnPc-A)**. In the cases of **14(**(*S*)**-H**_**2**_**Pc-B)** and **14(**(*S*)-**CuPc-B)**, the former H_2_Pc self-assembles into clockwise right-handed screw-like fibers (ca. 10 μm length, 6 μm width, and 1.5 μm helical pitch), while the latter CuPc self-assembly into anti-clockwise left-handed aggregates of fibers (ca. 25 μm length, 1 μm width, and 0.7 μm helical pitch) (Tian et al., 2013b). In the mesophase, **15(**(*S*)-**CuPc(αOC**_**5**_**H**_**11**_**)**_**4**_**)** gathers into rectangular columnar liquid crystals with anti-clockwise fan textured structure. However, from **14(**(*S*)-**CuPc(βOC**_**5**_**H**_**11**_**)**_**8**_**)**, hexagonal columnar liquid crystals with well-ordered, rare, clockwise spherulites were produced. The helical self-assembly and non-linear optical properties of (*S*)-**CoPc(αOC**_**5**_**H**_**11**_**)**_**8**_ were comparatively investigated with its analog having the same chiral moieties linked by substituents at β-positions, i.e., **15(**(*S*)-**CoPc(βOC**_**5**_**H**_**11**_**)**_**8**_**)** (Lin et al., 2015). Although these two compounds have the identical chiral functional group, they showed different CD signals in the Q absorption region of the corresponding complex, and gathered into different morphologies. At the initial stage, they got together into clockwise ribbon-like structures, while in the further later stage of assembly, they formed either clockwise or anti-clockwise fibrous structures.

## Chiral Subphthalocyanine Systems

**Figure 4:**
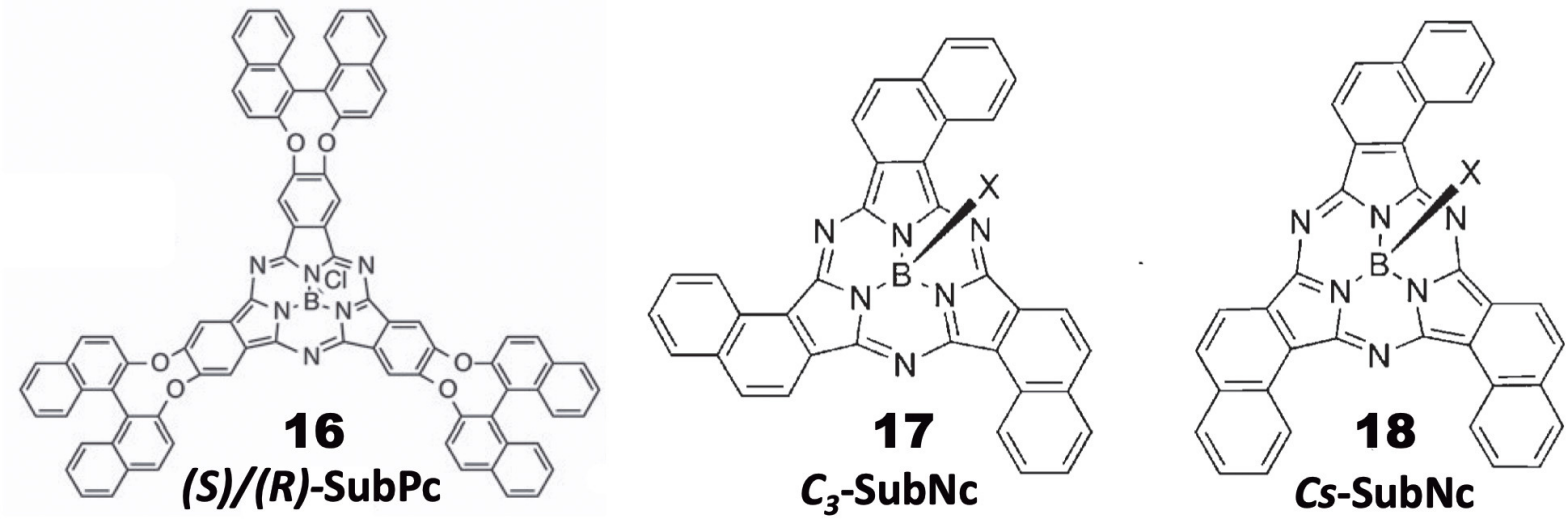
Structures of *(S)-* or *(R)-*binaphthyl-linked SubPc **16** and *C*_3_*-* **17** and *C*_*s*_*-*subnaphthalocyanine (SubNc) **18**.

General Pcs consist of four isoindoline units and are almost flat, but subphthalocyanine (SubPc) is a Pc congener consisting of three isoindoline units and boron coordinated with an axial ligand (Figure 4). Accordingly, it has a cone-shaped structure and blue-shifted Soret and Q bands. As a chiral SubPc, peripherally chiral binaphthyl-linked SubPc **16** has been known since 2014 (Zhao et al., 2014). The starting materials are *(S)-* and *(R)*-**a** in Figure 1. By refluxing these in the presence of BCl_3_ in 1,2,4-trichlorobenzene, the target SubPcs **16** were obtained in ca. 33% yield. Even the ^1^H-NMR spectra are interesting in that the ring-current effect is different between the *endo* and *exo* sides (the ring current of the SubPc ring is stronger in the concave side of the bowl than the convex side) (Shimizu et al., 2012). Thus, since one binaphthyl unit lies on the *exo* side while the other lies on the *endo* side, the ^1^H proton signals of binaphthyl appeared far away from each other.

Subnaphthalocyanine (SubNc) can be obtained by reacting naphthalonitrile in the presence of a boron compound, such as BCl_3_ in a solvent, such as dimethylbenzene or trimethylbenzene (Kobayashi, 2002). If 1,2-dicyanonaphthalonitrile is used as a starting material, the product is a mixture of inherently chiral ***C***_**3**_**-SubNc 17** and ***C***_***s***_**-SubNc 18** isomers, which can be separated by column chromatography. Further, each isomer can be resolved, by using chiral columns, to clockwise and anti-clockwise enantiomers viewing from the axial ligand (Shimizu et al., 2011).

**Figure 5:**
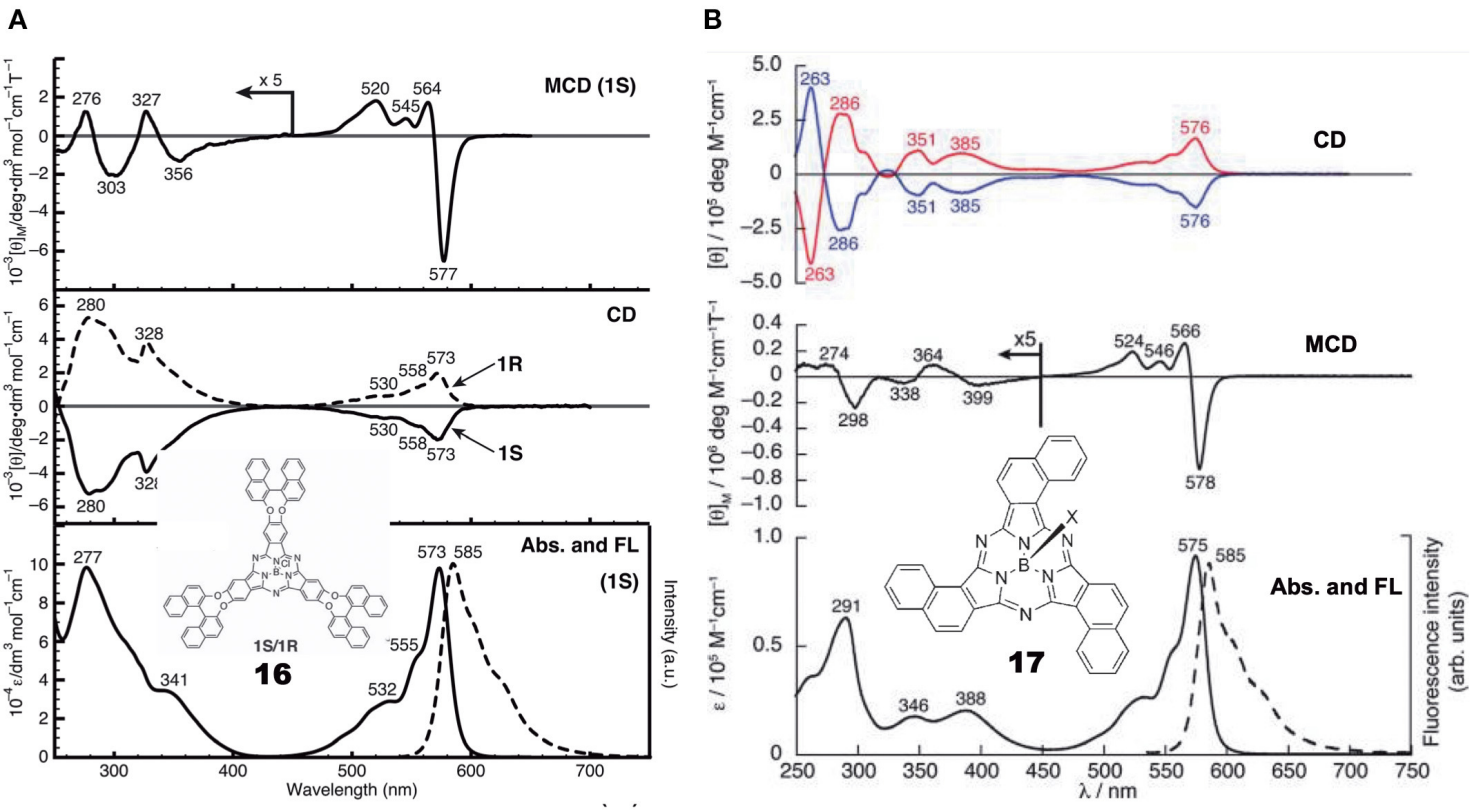
Electronic absorption, fluorescence, CD, and MCD spectra of **(A)** chiral binaphthyl-linked SubPc **16**, and **(B)** *C*_3_-SubNc **17**. In the CD of *C*_3_-SubNc **17**, the red and blue lines are from enantiomers whose naphthalene rings are arranged anti-clockwise and clockwise, respectively. Adapted from Shimizu et al. (2011) and Zhao et al. (2014).

Figure 5 shows the absorption, fluorescence, CD and MCD spectra of SubPc with chiral binaphthyl-linked SubPc and ***C***_**3**_**-SubNc 17**. The Q and Soret bands of SubPc generally appear at 570 and 280–300 nm, blue-shifted compared with those of Pcs [ca. 650–680 and 330–350 nm, respectively (Fukuda and Kobayashi, 2010)]. The Q-band MCD signals are mainly a contribution of Faraday C terms, since the SubPc chromophore itself can be approximated as having *C*_3*v*_ symmetry, i.e., the first excited state is doubly degenerate. The enantiomer with *(R)*- and *(S)*-chirality in the binaphthyl moiety produced plus and minus CD signs, respectively, over the whole window region. The angle between the two naphthyl units in binaphthyl is about 66 degrees, and the sign and intensity of CD can be reasonably explained similarly to the chiral binaphthyl-linked Pc systems (Kobayashi et al., 1999a), although the SubPc system might be slightly more difficult due to its bowl-shaped structure. Nevertheless, molecular orbital calculations using the TD-DFT method succeeded in reproducing the minus CD signals associated with the Q and Soret bands of the *(S)*-**SubPc 16** compound. The absorption spectrum (particularly wavelengths) of 1,2-SubNc does not differ significantly from that of SubPc, since outer benzene rings are not fused in a radial direction, viewing from the central boron of the molecule (Kobayashi and Konami, 1996). The two peaks on the longer-wavelength side of the Soret band are characteristic of naphthalocyanine derivatives (Kobayashi, 2002). Although not shown in this review, the absorption spectrum of the *C*_*s*_-isomer **18** is almost identical to that of the *C*_3_-isomer **17** in Figure 5B, so that the MCD spectra of the two isomers also have very similar band shape to each other. However, the CD intensity of these isomers are quite different, where that of the *C*_3_-isomer **17** is about three times stronger than for the *C*_*s*_-isomer **18**. Theoretically, the CD intensity (rotational strength) is directly proportionate to the product of the transition electric moment (μ) and the transition magnetic moment (*m*), i.e., μ*m*, whilst the absorption intensity (oscillator strength) is proportionate to the square of μ, i.e., μ^2^ (Kobayashi et al., 2012). The fact that the absorption spectra of the *C*_3_- **17** and *C*_*s*_-isomers **18** are almost identical means that the μ values of these two compounds are also considered almost identical. Therefore, we can attribute the CD intensity difference between the *C*_3_- **17** and *C*_*s*_-isomers **18** to *m*. The transition magnetic moment *m* is generated along the z-direction when charge is rotated in the x-y plane. If we compare the geometrical structures of the *C*_3_*-* **17** and *C*_*s*_-isomers **18** of 1,2-SubNcs, it sounds feasible that the net *m* values of the *C*_3_-isomer **17** are lessened to one-third in *C*_*s*_-isomer **18** by one oppositely-arranged naphthalene moiety in the latter isomer. Thus, this is a very rare but exemplary system which certifies that the magnitude of *m* is really parallels the rotation of charge. The absolute structure of *C*_3_-SubNcs **17** elucidated by X-ray crystallography led to a comprehensive understanding of the structure-chirality relationship of 1,2-SubNcs; i.e., the minus CD signals in the Q-band region indicate a molecular structure where the naphthalene moieties are arranged right-handed, whilst the plus CD signals in the same region indicate an left-handed arrangement viewing from the axial ligand side.

## Optically-Active Systems due to Coordination of Chiral Ligands

Many chromophores are not optically active, but by coordination of an extraneous chiral ligand, the system becomes optically active. Zhang et al. (2011) added equimolar amounts of chiral diamine to tetracarboxyphthalocyanine metal complexes (MtTCPc, Mt = Cu, Ni) in DMSO/CHCl_3_ mixed solvent, and succeeded in inducing CD signals in the region of the absorption spectrum. The diamines used were: (1*R*,2*R*)-(-)- and (1*S*,2*S*)-(+)-1,2-diaminocyclohexane, (1*R*,2*R*)-(+)- and (1*S*,2*S*)-(+)-1,2-diphenyl-1,2-ethylenediamine (DPEA), (*R*)-1,1′- and (*S*)-1,1′ -binaphthyl-2,2′-diamine, in addition to (*R*)-1-phenylethanamine, (*R*)-1-(naphthalene-1-yl)-ethanamine, and (-)-sparteine. The CD signal induced by adding (1*R*,2*R*)- and (1*S*,2*S*)-diamine exhibited a mirror-symmetry structure in relation to the signal intensity = zero line, and its intensity was largest when the amount of diamine was equimolar to that of MtTCPc. By increasing CHCl_3_ (poor solvent) in DMSO (good solvent) from 1:1 (v/v), the absorption spectrum changed to that of cofacial aggregates, and concomitantly the CD intensity decreased. Surprisingly, in the presence of *(R)-* or *(S)*-DPEA, MtTCPc (M = Ni, Cu) showed no significant CD and UV/Vis spectral changes before and after the addition of an equimolar amount of the corresponding antipode (*(S)-* or *(R)*-DPEA, totally, *R/S* or *S/R* = 1:1), implying a very quick self-gathering ability of MtTCPc that produces highly stable optically-active MtTCPc π–π stacks whose chirality is immobilized firmly.

The aggregate's morphology of supramolecular MtTCPc was studied by high resolution TEM. Samples on a carbon-coated copper minigrid were prepared by casting a drop of MtTCPc solution onto its surface. This showed that supramolecular MtTCPcs (M = Ni, Cu) adopted an entangled structure composed of nano-structural fibers of about 10 nm diameter and 150–700 nm length.

## Chiral Congeners of Phthalocyanine-Related Compounds

**Figure 6:**
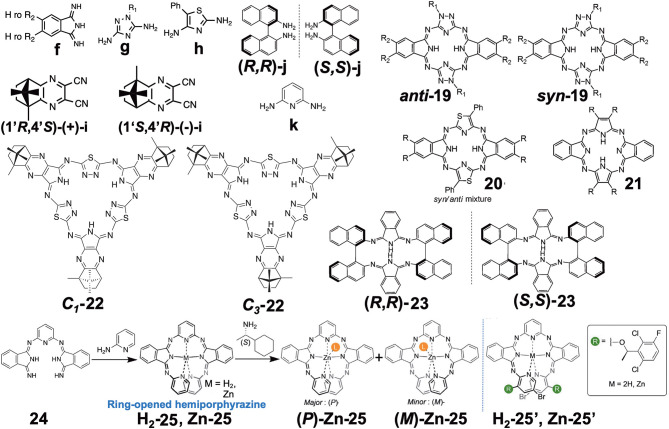
Various types of optically-active hemiporphyrazine derivative reported in the 2010–2020 period.

Four papers reporting various types of hemiporphyrazine (Figure 6) were found in this category, and they are introduced in chronological order. ***anti*-19** and ***syn*-19** were prepared at a ratio of ca. 1:1 by heating an equimolar amount of isoindolediimine **f** and triazole diamine **g** (Muranaka et al., 2014). *anti* and *syn* structures were confirmed from ^1^H-NMR and IR spectra: The pyrrole proton signal appeared at around 15 ppm, but as a singlet for ***anti*-19** and two singlets for ***syn*-19**, and in the powder state, the former showed a single NH stretching vibration at 3,298 cm^−1^, while two peaks were detected for the latter at 3,363 and 3,291 cm^−1^. Insertion of VO to ***anti*-19** was performed in order to obtain intrinsically-chiral triazolehemiporphyrazine (Kobayashi et al., 2009), whose resolution was achieved by high-performance liquid chromatography. In order to compare the properties of **19** with those of congeners, **20** and **21** were synthesized from isoindolediimine **f** and thiazole diamine **h** in 2-ethoxyethanol at 135°C, and isoindolediimine **f** and 4,5-dicyano-4-octene in the presence of magnesium in butanol at boiling temperature, respectively. Pyrrole proton ^1^H NMR signals of **20** and **21** were observed at ca. 13.6–14.2 and −1.93 ppm, respectively, suggesting that **20** is anti-aromatic while **21** is aromatic.

**Figure 7:**
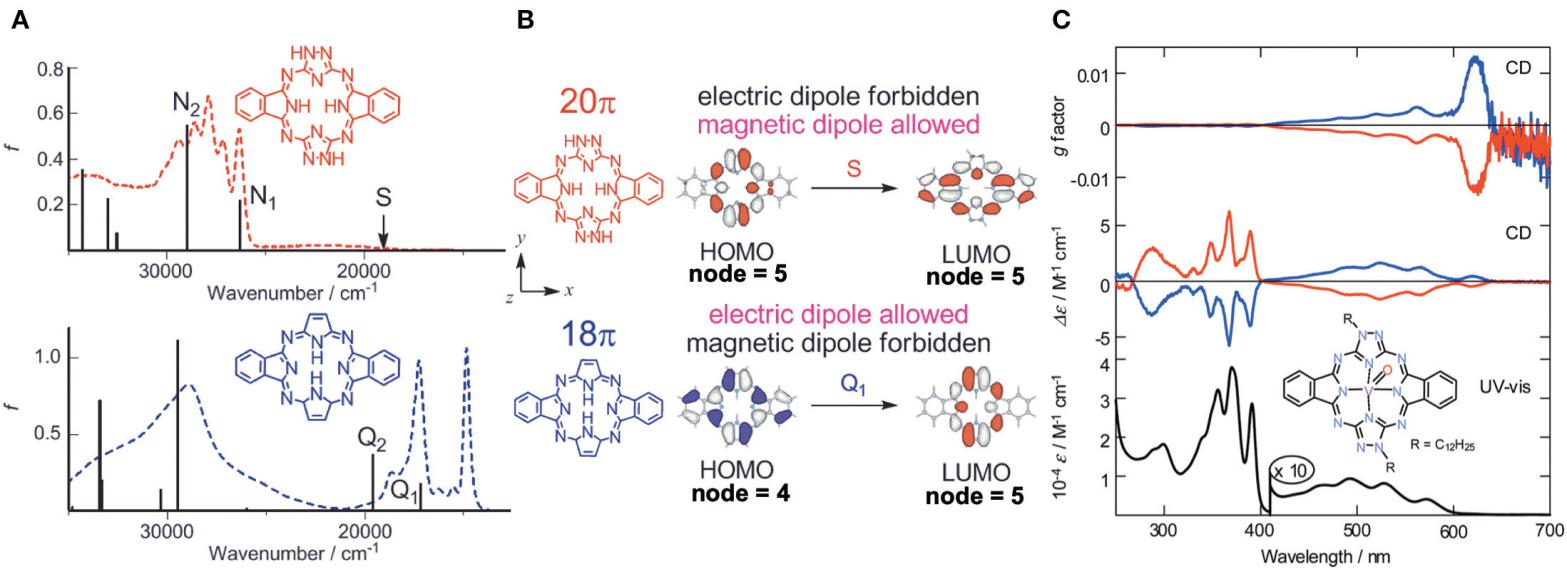
**(A)** Electronic absorption spectra of ***anti*-19** (top) and **21** (bottom), together with calculated absorption spectra. **(B)** HOMO and LUMO of 20π- (top) and 18π porphyrinoid systems (bottom). **(C)** Electronic absorption, CD, and CD (anisotropy g factor profile) spectra of the VO complex of ***anti*-19**. According to the theoretical calculations, the blue lines correspond to data of the anti-clockwise enantiomer whose structure is drawn inside. Adapted from Muranaka et al. (2014).

Molecular orbitals (MOs) of these molecules were calculated, and it was found that the *anti*-forms of **19** and **20** have HOMO and LUMO with five nodal patterns, while those of **21** have four and five nodal patterns, respectively. Obviously, the nodal patterns of the HOMO−1, HOMO, and LUMO of the ***anti-*19** and **20** were respectively in connection with the HOMO, LUMO, and LUMO+1 of the 18π-electron dibenzotetraazaporphyrin **21**. The LUMO and HOMO of **19** and **20** are derived from a pair of degenerate LUMOs of the 18π-electron perimeter model, while from one of the degenerate HOMOs, the HOMO−1 is derived. Accordingly, together with the pyrrole proton ^1^H NMR data at very low field, **19** and **20** were judged to be 20π anti-aromatic [4*n*π]-electron systems. This kind of system can be analyzed using Michl's perimeter model (Fleischhauer et al., 2000, 2004, 2005; Muranaka et al., 2009). Figure 7A shows the experimental and calculated absorption spectra of ***anti-*19** and **21**. The spectral pattern of **21** retains characteristic features of a low-symmetrical tetraazaporphyrin with *D*_2*h*_ symmetry (Kobayashi and Konami, 1996; Kobayashi, 2002), showing two prominent, split Q envelopes with coupled Faraday B-terms in the visible region (not shown). ***anti*-19**, on the other hand, revealed a weak absorption band with a pronounced vibronic progression in ca. 400–600 nm region, followed by stronger bands in the UV region of <400 nm. If we pay attention to the HOMO-LUMO transition, it is an intrashell ΔM_L_ = 0 forbidden transition for ***anti-*19** and an ΔM_L_ = ±1 allowed transition for **21**, so that the longest wavelength region of ***anti-*19** is very weak, while **21** has some intensity. Indeed, as seen in Figure 7B, the HOMO-LUMO transition accompanies a rotation of charge for a 20π system (magnetic dipole allowed), but a translation of charge for 18 a π system (electric dipole allowed). The spectra of two enantiomers of the VO complex of ***anti*-19** are collected in Figure 7C. Most notably, a large g-factor associated with the electric dipole forbidden band in the 400–650 nm region was detected. The value of g-factor associated with the lowest-energy S band at ca. 615–620 nm was larger than 0.01, in accord with the rotation of charge produced by intrashell forbidden transitions. This was the first report of unambiguous spectral fact that cyclic [4*n*π]-electron molecules have indeed a large transition magnetic dipole moment in the region of the lowest π-π^*^ transition with forbidden character. The g-factor spectrum ends at ca. 420 nm, where the electric dipole allowed transitions start to appear.

Filatov et al. reported the so-called 3+3 compound **22** in 2014 (Filatov et al., 2014). The starting compounds, **(1′R,4′S)-(+)-i** and **(1′S,4′R)-(**–**)-i** were synthesized by reacting (1*S*)-(+)- or (1*R*)-(–)-camphorquinone and diaminomaleonitrile in acetic acid at 50°C (Kobayashi and Nevin, 1998). Compound **22** was obtained by heating **(1′R,4′S)-(+)-i** and **(1′S,4′R)-(**–**)-i** together with 2,5-diamino-1,3,4-thiadiazole in either butanol in the presence of CH_3_ONa at ca. 100°C (yield 10%) or in ethylene glycol in the absence of CH_3_ONa at refluxing temperature (yield 30%). Separation of *C*_1_ and *C*_3_ symmetry regioisomers was carried out using chiral columns. The ratio of ragioisomer, *C*_1_:*C*_3_ was 57:43 for the reaction in butanol and 76:24 in ethylene glycol. In ^1^H NMR spectra, the pyrrole NH signal of the *C*_3_ isomer appeared as a singlet at 12.44 ppm, and as three singlets at 12.45, 12.43, and 12.42 ppm for the *C*_1_ isomer, confirming an insignificant, if any, diatomic ring current.

**Figure 8:**
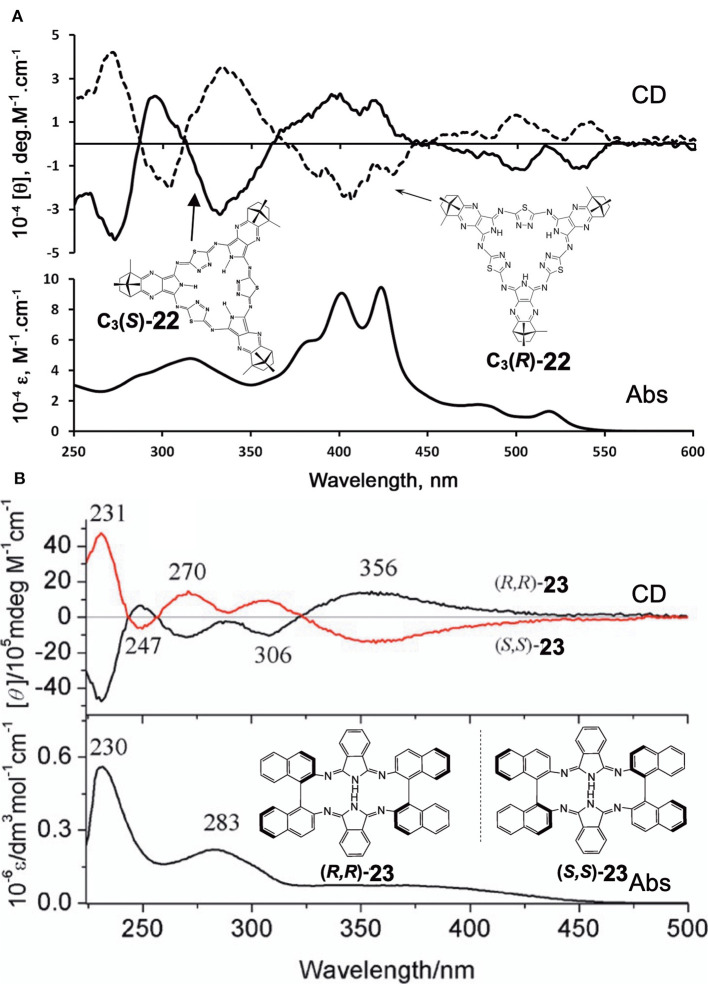
Electronic absorption and MCD spectra of **(A)** ***C***_**3**_**(*S*)**- and ***C***_**3**_**(*R*)*-*22** and **(B) (*R,R*)-23** and **(*S,S*)-23**. Adapted from Filatov et al. (2014) and Wu et al. (2016).

Figure 8A shows the absorption and CD spectra of **22**. Absorption peaks appeared at 515, 474, 421, and 399 nm, of which two peaks at longer wavelength are weak, while those at shorter wavelength are intense. According to MO calculations recently performed, these are 30π-electron, 4n+2 aromatic systems (Bacilla et al., 2020). Both the HOMO and LUMO are doubly degenerate, so that these are grouped into “double-soft chromophore” systems (Michl, 1978a,b,c; Michl, 1980). The two weak bands at longer wavelength correspond to transitions from M_L_ = −7 to +8 and 7 to −8 (i.e., ΔM_L_ = ±15) transitions with forbidden character, whereas the intense bands in the ca. 380–440 nm correspond to allowed transitions with ΔM_L_ = ±1. Concerning the CD spectra of **C**_**3**_**(*S*)**- and **C**_**3**_**-(*R*)-22** which exhibit a mirror-symmetry pattern in relation to the [θ] = 0 line, no interpretation was attempted. Since CD in this system is generated only by optically-active carbons, its shape and intensity cannot be explained at least using the concept of CD (Kobayashi et al., 2012).

**Figure 9:**
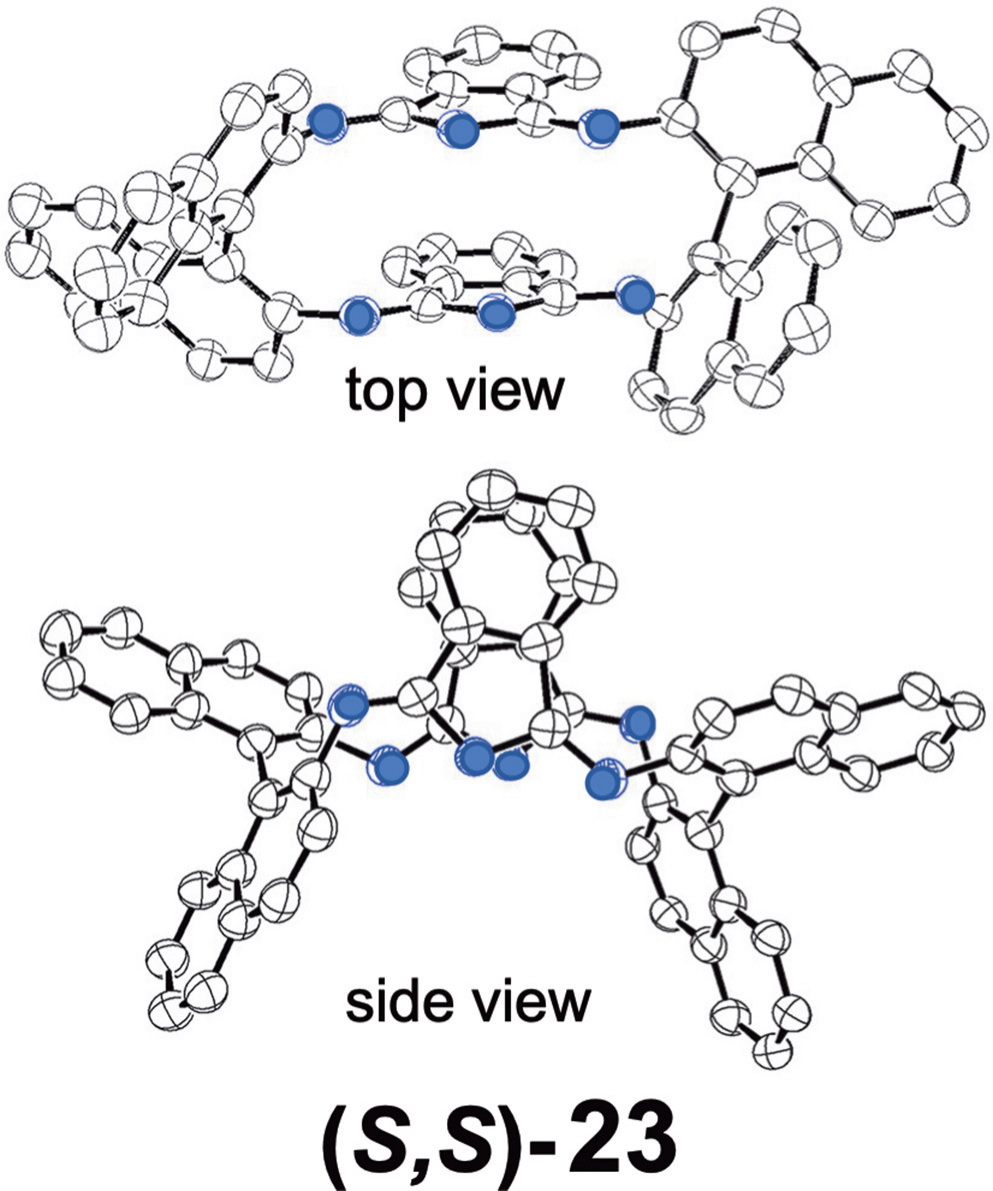
X-ray crystal structure of **(*S,S*)-23**, top view and side view. Adapted from Wu et al. (2016). Blue circles indicate nitrogen.

Shen et al. reported compound **23**, which is a kind of hemiporphyrazine that was prepared via core modification of the macrocycle adopting an intrinsically chiral ring molecule as a part of the inner perimeter of the macrocycle (Wu et al., 2016). A condensation reaction of enantiopure chiral diamine, **(*R,R*)-j** or **(*S,S*)-j** and isoindolediimine **f** was performed in *n*-butanol at 150°C for 12 h to give the **(*R,R*)-23** and **(*S,S*)-23** target compounds as yellow solids in ca. 3–7% yield. Although their mass and ^1^H NMR data were in accordance with the structure shown in Figure 8, single crystal X-ray analysis further confirmed the structure of **(*S,S*)-23**, where the two naphthyl moieties lie around a pseudo *C*_2_ axis and are inflexible, with an angle of ca. 85 degrees. The two isoindole moieties at opposite positions lie parallel to each other forming a kite-like conformation, so that the two nitrogen atoms in the isoindoline moiety point in the same direction (Figure 9). Thus, although previously reported himiporphyrazines have roughly planar structures, in compounds **23**, all constituting chromophores are quite strongly deviated from the inner perimeter plane, but still exhibit absorption bands in the longer-wavelength region (ca. 260–500 nm) that the constituting chromophore units do not show (Figure 8B). Here, in the absorption spectra, a broad intense band is seen at 283 nm, in addition to a weaker band envelope in the 325–500 nm region, which is in agreement with what is generally observed for non-aromatic hemiporphyrazines. **(*R,R*)-23** and **(*S,S*)-23** molecules contain 18 atoms and 20-π-electrons along their inner perimeter, so the authors inferred that a Mobius strip type structure could conceivably result in Mobius aromaticity. Since the isoindoline and binaphthyl moieties are severely deviated from planarity of the macrocycle, the calculated frontier π MOs of **23** were predicted to be significantly localized on either the 1,1′-binaphthyl or isoindoline moieties, so that the spectral bands could not be easily assigned using Michl's 4N perimeter model (Howeler et al., 1998; Fleischhauer et al., 2000, 2004, 2005). Nevertheless, both the CD and absorption spectra predicted by TD-DFT calculations on the X-ray structure of **(*S,S*)-23**, were in close agreement with those recorded experimentally.

Muranaka and Uchiyama et al. recently reported ring-opened helical hemiporphyrazines, **H**_**2**_**-25 and Zn-25**, exhibiting circularly-polarized luminescence (CPL) (Tanaka et al., 2020). CPL-active compounds have attracted the attention of scientists, not only due to purely scientific interest, but also due to their potential applications. In order to obtain strong CPL signals accompanying a high luminescence dissymmetry factor (g_lum_), it is important to design molecules with a large transition magnetic dipole moment (*m*) compared to the transition electric dipole moment (μ). Since many fluorescent organic molecules have a small *m* and large μ for the S_1_-S_0_ transition, their g_lum_ is low (g_lum_ = 10^−5^-10^−3^). This is quite different from the *f-f* transition in lanthanide metal complexes and the π^*^-*n* (S_1_-S_0_) transition in carbonyl-containing compounds, since they have an almost zero μ and a large *m* (Riehl and Richardson, 1986; Lunkley et al., 2008). The latter case led to a relatively high luminescence dissymmetry factor (g_lum_ = 10^−3^-10^0^), but unfortunately in these systems, the emission quantum yield is very low due to the very small μ. If we design hemiporphyrazine which is considered to be a 20π system, the absorption at the longest wavelength accompanies rotation of charge, as shown in Figure 7B, meaning that the *m* of this transition is large. However, in order to obtain stronger fluorescence and CD spectra, an intense μ is also desirable. Accordingly, planar molecules do not generally satisfy these two requirements. Compounds **H**_**2**_**-25** and **Zn-25** are helical molecules due to steric interaction of the pyridine moiety, so that they may satisfy these requirements after resolution of the enantiomers. Their precursor **24** was first prepared by reacting 2,6-diaminopyridine and substituent-free isoindolinediimine **f** at a ratio of 1:2, which was then reacted with two equivalents of 2-aminopyridine, to afford **H**_**2**_**-25**. Resolution of **H**_**2**_**-25** into its enantiomers was unsuccessful, but after insertion of a Zn ion, **Zn-25** was resolved into its *P*- and *M-*enantiomers at 0°C, due to relatively high solubility in a range of solvents. However, at room temperature, the enantiomers were racemized immediately due to a low energy barrier for racemization.

**Figure 10:**
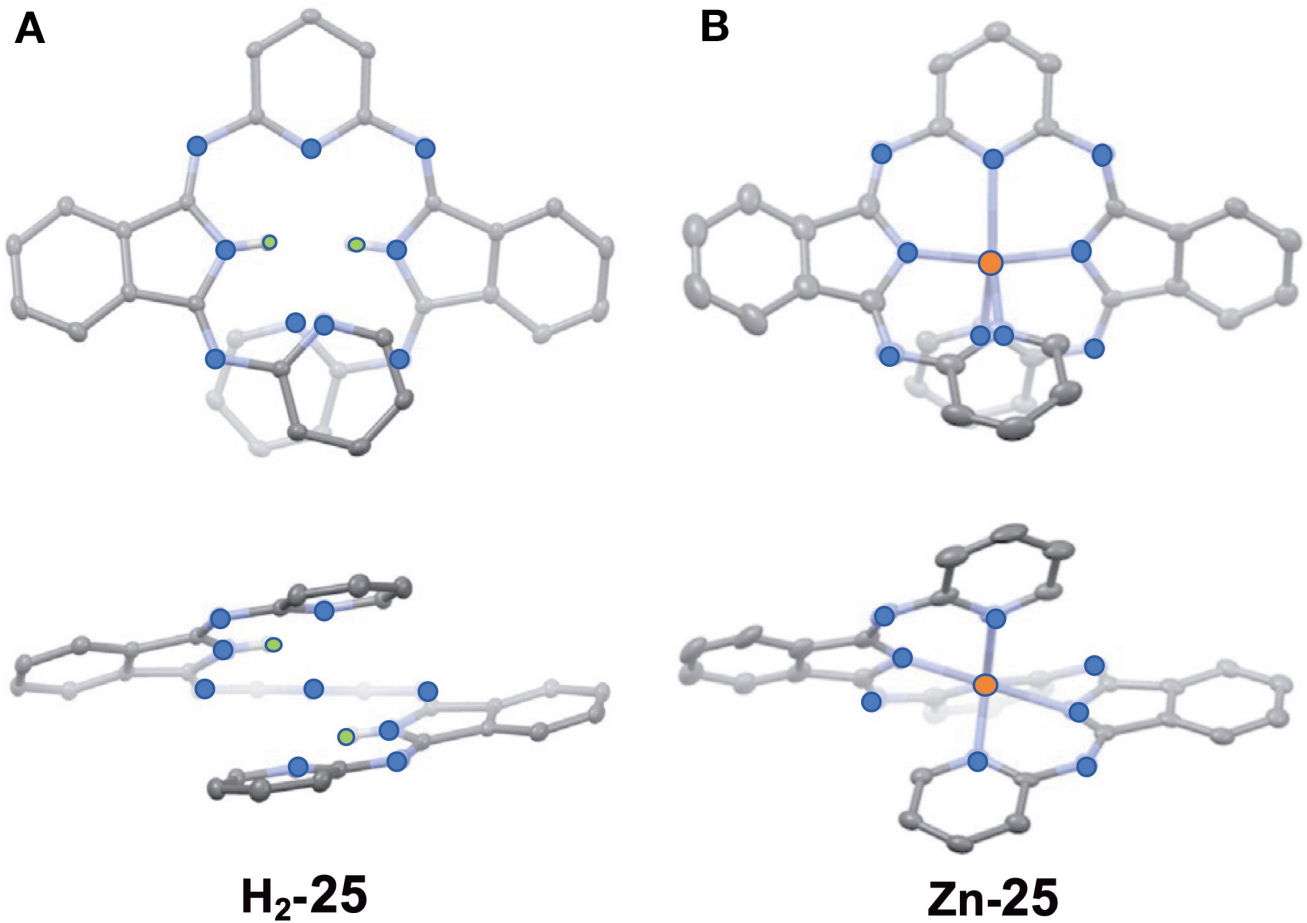
X-ray structures of **H**_**2**_**-25 (A)** and **Zn-25 (B)**. The thermal ellipsoids are scaled to the 50% probability level. Hydrogen atoms except for NH hydrogens in **H**_**2**_**-25** are omitted for clarity. One of the two enantiomers is shown. Blue-, orange, and green circles indicate nitrogen, zinc, and hydrogen, respectively. Adapted from Tanaka et al. (2020).

Figure 10 shows the X-ray structure of one enantiomer of each of **H**_**2**_**-25** and **Zn-25**, which have helical geometry due to steric repulsion between the pyridine moieties. All five nitrogen atoms in the pyridine and isoindoline moieties coordinate to the Zn(II) ion so that the central part is in a distorted trigonal bipyramidal geometry.

**Figure 11:**
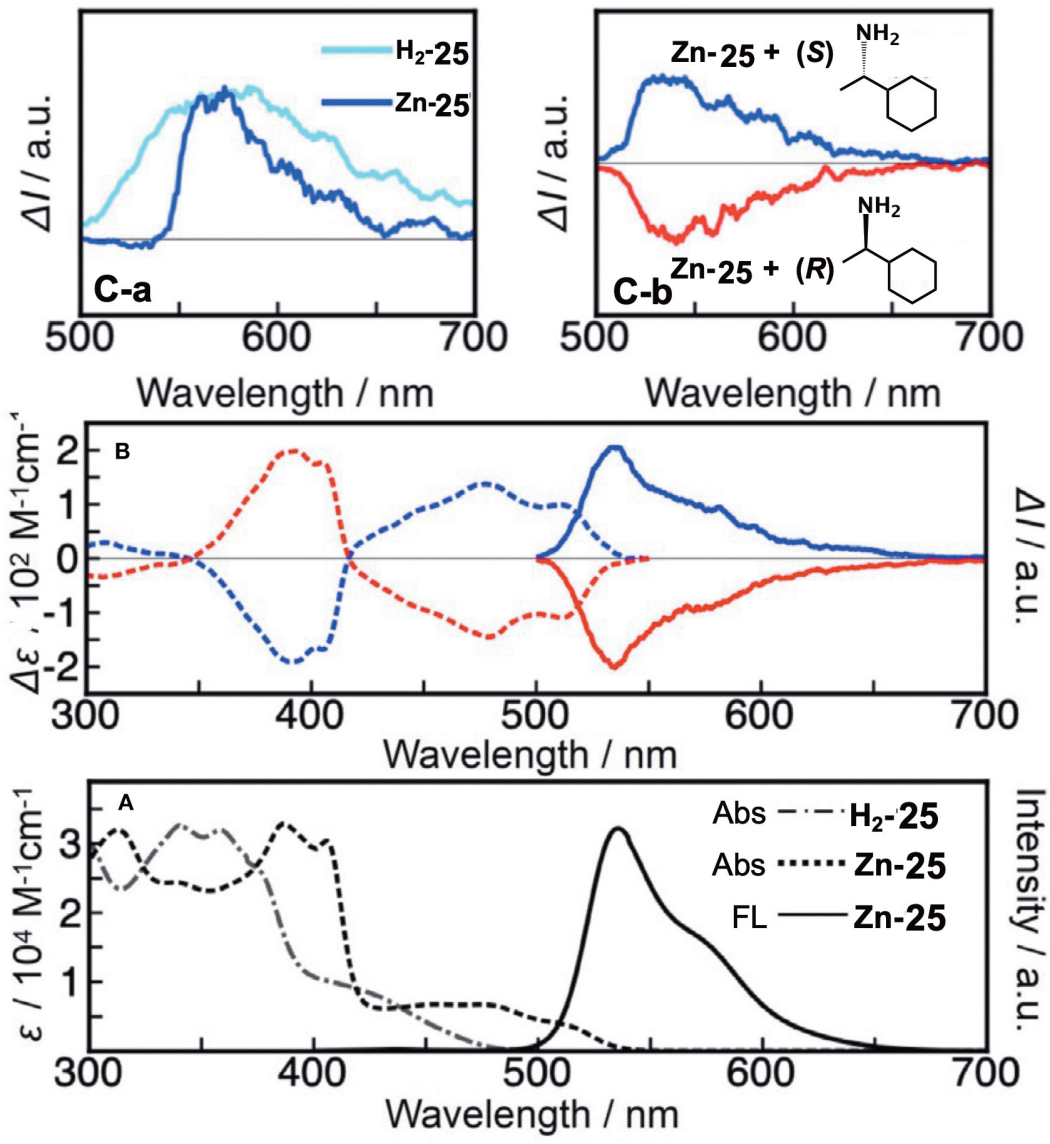
**(A)** Electronic absorption and fluorescence spectra of **H**_**2**_**-25** and **Zn-25** in CH_2_Cl_2_ at ambient temperature. **(B)** CD and CPL signals of **Zn-25** in *n*-hexane/THF/diethylamine = 80:20:0.1 (v/v/v) (first fraction, blue line; second fraction, red line). According to the TDDFT calculations, the first and second fractions were respectively assigned as right-handed (*P*)- and left-handed (*M*)-**Zn-25**. **(C-a)** CPL signals of ring-unclosed hemiporphyrazines bearing chiral units (**H**_**2**_**-25′** in toluene and **Zn-25′** in CHCl_3_) at ambient temperature. **(C-b)** CPL signals of **Zn-25** in the presence of (*S*)- or (*R*)-1-cyclohexylethylamine.

Figure 11A shows the absorption spectra of **H**_**2**_**-25** and **Zn-25**. **H**_**2**_**-25** exhibited a shoulder absorption at 380–500 nm, and a similar weak band appeared for **Zn-25** in 420–540 nm which could be assigned to a (S_0_-S_1_) π-π^*^ transition (Muranaka et al., 2014). The molar extinction coefficient of **H**_**2**_**-25** at 430 nm was ca. 3–4 times larger than that of conventional hemiporphyrazines, plausibly by ring-opening, suggesting that μ became larger. The calculated absorption spectra also support the observed spectra to a fair extent, and the oscillator strengths for the S_0_-S_1_ transition of **H**_**2**_**-25** and **Zn-25** were 0.16 and 0.11, respectively, in comparison with 0.00 for hemiporphyrazine. As shown in Figure 11B, resolved enantiomers of **Zn-25** exhibited CD and CPL envelopes of mirror symmetry in connection to the intensity = 0 line. The g_lum_ value of **Zn-25** at 535 nm was estimated by calculation to be ±2.1 × 10^−2^, representing one of the largest values among to date reported small helical molecules (Sanchez-Carnerero et al., 2015). The value of absorbance dissymmetry factor, g_abs_ = ±2.6 × 10^−2^, obtained experimentally from CD spectra at 520 nm, was very close to the g_lum_ value in CPL spectra, suggesting that structural change was small upon excitation.

In order to elucidate the origin of the CPL properties, they performed MO analysis for the optimized S_1_ state geometry of **Zn-25** and hemiporphyrazine zinc complex as a reference. The properties of the associated HOMOs and LUMOs, such as coefficient values and nodal patterns and numbers, were retained even after structural modification to the helical geometry, and the estimated values of transition magnetic dipole moment (*m*) for the S_0_-S_1_ transition of (*P*)-**Zn-25** was 3.64 au, which was close to that of zinc hemiporphyrazine (4.35 au). The large g_lum_ value for **Zn-25** was, therefore, taken to be related to the peculiar molecular orbital feature of the ring-opened hemiporphyrazines.

A rigid CPL-active ring-unclosed hemiporphyrazine (**H2-25′**) and its zinc complex (**Zn-25′**) (Figure 6) were also synthesized by using 2-aminopyridine accompanying a chiral structure. As seen in Figure 11C-a, compounds **H2-25′** and **Zn-25′** showed positive CPL spectra at ambient temperature without chiral HPLC separation (**H2-25′**: g_lum_ = +6.5 × 10^−3^ at 587 nm in toluene; **Zn-25′**: g_lum_ = +1.0 × 10^−2^ at 573 nm in CHCl_3_), indicating that the ring-unclosed hemiporphyrazines with the right-handed (*P*)-helical structure were successfully obtained in a diastereoselective manner by covalently linking the chiral subgroup to the skeleton. The chiral recognition ability of (*R*)- and (*S*)-**Zn-25** was then examined by means of CPL spectroscopy since they racemized rapidly at ambient temperature. Here, when (*R*)- or (*S*)-cyclohexylamine was added to a CH_2_Cl_2_ solution of **Zn-25**, a minus or plus CPL signal with a g_lum_ value of ±1.3 × 10^−3^ at 541 nm was recorded (Figure 11C-b), depending on the chirality of amine. Afterall, this phenomenon could be rationalized by means of the induction of helical chirality via ligation of the chiral amine to the central zinc ion. Thus, since the helical structure of **Zn-25** is flexible, it can be utilized as a dynamic chiral recognition/sensing tool.

## Conclusions and Outlook

Optically-active Pcs and their congeners are expected to be the subject of ongoing reports. As seen in this review, those containing chiral binaphthyls may become an important topic in future publications, for several conceivable reasons: easy and high-yield preparation of precursors from commercially available compounds, i. e. **a** and **b** in Figure 1; no racemization of **a** and **b** during macrocyclic formation reaction; and anticipated strong CD and ICD intensity and relatively easy theoretical analysis of the spectroscopic properties of the resultant compounds. In particular, since enantiopure (*R,R*)- and (*S,S*)-2,2′-dihydroxybinaphthyl are commercially available, researchers will continue to use these precursors often. Since the synthesis and CD properties of normal compounds are no longer uniquely interesting, because two enantiomers always afford CD of mirror image with respect to the [θ] = 0 line, it is important to produce compounds which have spectroscopically intriguing properties, and to analyze the data theoretically if needed with the help of quantum chemical calculations. In most papers including chiral binaphthyl units published over the past decade, however, no analysis of CD spectra has been given, unfortunately.

The studies of Pc derivatives substituted by long alkyl chains have mostly been related to liquid crystals. Interestingly, it was found and established by Ohta et al. that some of the achiral dyads based on Pc and fullerene (Pc-C_60_) show a unique helical structure in the liquid crystalline phases (= mesophases) (Tauchi et al., 2010). However, a problem is that some researchers did not have enough expertise on the mesophase structural analysis. For example, a paper from a high-level university reported that other Pc-C_60_ based-dyads exhibit the helicity, although their assigned mesophase cannot show theoretically a homeotropic alignment necessary for the establishment of the helicity (Hayashi et al., 2011, 2017). The authors assigned a faint shoulder in a diagram of temperature-variable X-ray diffraction measurements (XRD pattern) to the helicity, but the homeotropic alignment should not appear theoretically for their assigned mesophase. This assignment was later shown to be false in a detailed study using compounds having similar structures by a group of liquid crystal specialists (Ishikawa et al., 2018). Thus, researchers of liquid crystals are recommended to learn more about the analysis of mesophase data. In this respect, a nice textbook was published recently (Ohta, 2020).

In the work introduced here as Pc congeners, for example, Muranaka's **19–21** were interesting. Although the structures were similar, depending on the element included, the spectra changed between those of 4*n*π and (4*n*+2)π systems, and in the case of the VO complex of 4*n*π ***anti*-19**, the two enantiomers showed an intense CD in the region where rotation of charge is expected theoretically. This became possible since in 4*n*π compounds, the HOMO-LUMO transition has essentially an intrashell nature (magnetic dipole allowed transition), and this kind of theme cannot be easily pursued without having some knowledge on spectroscopy. Rather than Pc itself, attractive compounds may be found more easily in Pc-related congeners, since structural modification possibilities are huge, and new ideas can come easily from researchers who always seek for fantastic azamacrocycles. In addition, the formation of some achiral dyad and triad systems that form helicity or chirality appears to be a challenging theme to be pursued going forward, in order to elucidate its driving force.

## Author Contributions

YO prepared the figures. TH collected the reference papers. NK wrote the text. All authors contributed to the article and approved the submitted version.

## Conflict of Interest

The authors declare that the research was conducted in the absence of any commercial or financial relationships that could be construed as a potential conflict of interest.
